# Presence of Antibodies Against *Coxiella burnetii* and Risk of Spontaneous Abortion: A Nested Case-Control Study

**DOI:** 10.1371/journal.pone.0031909

**Published:** 2012-02-21

**Authors:** Stine Yde Nielsen, Niels Henrik Hjøllund, Anne-Marie Nybo Andersen, Tine Brink Henriksen, Bjørn Kantsø, Karen Angeliki Krogfelt, Kåre Mølbak

**Affiliations:** 1 Department of Occupational Medicine, Regional Hospital West Jutland, Herning, Denmark; 2 Perinatal Epidemiology Research Unit, Aarhus University Hospital Skejby, Aarhus, Denmark; 3 Department of Clinical Epidemiology, Aarhus University Hospital, Aarhus, Denmark; 4 Department of Public Health, University of Copenhagen, Copenhagen, Denmark; 5 Department of Pediatrics, Aarhus University Hospital Skejby, Aarhus, Denmark; 6 Department of Microbiological Diagnostics, Statens Serum Institut, Copenhagen, Denmark; 7 Department of Microbiological Surveillance and Research, Statens Serum Institut, Copenhagen, Denmark; 8 Department of Epidemiology, Statens Serum Institut, Copenhagen, Denmark; The Australian National University, Australia

## Abstract

**Background and Aims:**

Q fever is a bacterial zoonosis caused by infection with *Coxiella burnetii*. It is well established that Q fever causes fetal loss in small ruminants. The suspicion has been raised that pregnant women may also experience adverse pregnancy outcome when the infection is acquired or reactivated during pregnancy. The purpose of this study was to assess the potential association between serologic markers of infection with *C.burnetii* and spontaneous abortion.

**Methods:**

A nested case-control study within the Danish National Birth Cohort, a cohort of 100,418 pregnancies recruited from 1996–2002. Women were recruited in first trimester of pregnancy and followed prospectively. Median gestational age at enrolment was 8 weeks (25 and 75 percentiles: 7 weeks; 10 weeks). During pregnancy, a blood sample was collected at gestational week 6–12 and stored in a bio bank. For this study, a case sample of 218 pregnancies was drawn randomly among the pregnancies in the cohort which ended with a miscarriage before 22 gestational weeks, and a reference group of 482 pregnancies was selected in a random fashion among all pregnancies in the cohort. From these pregnancies, serum samples were screened for antibodies against *C. burnetii* in a commercial enzyme-linked immunosorbent assay (ELISA). Samples that proved IgG or IgM antibody positive were subsequently confirmatory tested by an immunofluorescence (IFA) test.

**Results:**

Among cases, 11 (5%) were *C. burnetii* positive in ELISA of which one was confirmed in the IFA assay compared to 29 (6%) ELISA positive and 3 IFA confirmed in the random sample.

**Conclusions:**

We found no evidence of a higher prevalence of *C.burnetii* antibodies in serum samples from women who later miscarried and the present study does not indicate a major association between Q fever infection and spontaneous abortion in humans. Very early first trimester abortions were, however, not included in the study.

## Introduction

Q fever, a zoonotic infection caused by *Coxiella burnetii*, has previously been considered a rare, imported infection in Denmark, but recent studies have found antibodies against *C.burnetii* in a large percentage of Danish dairy herds and among individuals exposed to livestock animals [1]–[3].

In cattle and small ruminants Q fever is known to cause abortions, retained placenta, endometritis and infertility, and placentas of infected animals contain a high number of organisms [4], [5]. The bacteria remain viable for months in the environment and the most important route of transmission to humans is inhalation of contaminated aerosols.

For otherwise healthy people, Q fever infection is often asymptomatic or with a mild, flu-like course, but may also cause severe pneumonia. Pregnant women, immunocompromised patients and patients with pre-existing cardiac valve or vascular defects are at risk of a severe course of the infection [6], [7], [8].

The precise mechanisms by which the infection compromises pregnancy are largely unknown, but adverse pregnancy outcome has been reproduced in BALB/c mice in which infection followed by repeated pregnancies resulted in spontaneous abortion and perinatal death [9].

*C. burnetii* is an intracellular pathogen, but the cell types infected by *C.burnetii* in humans are unknown. A recent study used a human trophoblast cell line and found that *C.burnetii* infected and replicated within trophoblastic cells but the bacteria seemed unable to interfere with development of a normal pregnancy.

The study suggested that normal development of pregnancy may be impaired by the cooperation of trophoblasts and placental immune cells responsive to *C.burnetii* within the placental tissue [10].

Present evidence mainly originates from French case studies of referred pregnant women in which infection resulted in spontaneous abortion, intrauterine growth retardation, oligohydramnion, stillbirth and premature delivery in untreated pregnancies. One series of 53 cases demonstrated obstetric complications in 81% of Q fever positive cases not receiving long-term antibiotic treatment [11]. Infection in pregnancy is often asymptomatic but may imply an increased risk of chronic infection [8]. A risk of reactivation of a past infection in subsequent pregnancies has been described and infection in 1^st^ trimester may constitute a specific risk of spontaneous abortion [11]–[13].

Due to the sparse literature on Q fever in pregnancy, unbiased estimates of the risks of adverse pregnancy outcome among infected women remain largely unknown, and even though Q fever is endemic worldwide many obstetricians know little about the infection. The incidence of Q fever among pregnant women may therefore be underestimated [8].

The objective of the present study was to compare the prevalence of antibodies to *C.burnetii* in a random sample of pregnancies terminated by spontaneous abortion to the prevalence in the background population.

## Materials and Methods

### Ethics statement

Women enrolled in the Danish National Birth Cohort gave both verbal and written consent to participate. The women gave permission to include interview information, blood samples and health information from other registers in the Danish National Birth Cohort. The study was approved by the Danish National Birth Cohort, the Danish Data Protection Board, and the Danish Regional Scientific Ethical Committee.

### Participants

The study was based on interview data and blood samples from the Danish National Birth Cohort (DNBC), which is a nationwide cohort of 100,418 pregnant women and their offspring.

Enrolment in the DNBC took place between 1996 and 2002, and the women were recruited in connection with the first antenatal visit to the general practitioner. Gestational age at enrolment was scheduled to be 10 weeks. The median gestational week of enrolment was 8 weeks (25 and 75 percentiles: 7 weeks; 10 weeks), but some women were enrolled as early as in week 4 and as late as gestational week 27.

The percentage of pregnancies that resulted in a spontaneous abortion in the entire cohort was 4.7%. Foetal life table analysis has estimated the proportion of spontaneous abortions from gestational week 6 to be 11% in the DNBC [14].

Information on exposures before and during the early part of pregnancy was collected by means of a computer assisted telephone interview scheduled to take place in gestational week 12 or as soon as possible thereafter. In case of fetal loss before this interview, participants were offered a similar interview as soon as possible after the fetal loss (interview forms available at www.bsmb.dk).

During pregnancy, two blood samples were collected; one around gestational week 6–12, the second in gestational week 24. A sample was also drawn from the umbilical chord.

The interviews were not performed if the women were not reached within four phone calls, or did not wish to participate.

A more detailed description of the cohort can be found elsewhere [15].

This study was designed as a nested case-control study. A number of 200 pregnancies were randomly selected from the 4740 participants who experienced a miscarriage before 154 gestational days (22 gestational weeks), and for whom a serum sample was taken at the first antenatal visit at the GP and stored in a bio bank.

The case definition was miscarriage, defined as fetal loss before 154 days (22 weeks) after the self-reported first day of the last menstrual period.

A base sample of 500 non-cases was randomly selected among the 92500 participants with an existing first blood sample from early pregnancy. A total of 18 of the pregnancies in the base sample had spontaneous abortion as outcome and were consequently reclassified as cases.

The random selection of cases and non-cases irrespectively of participation in the scheduled interviews was chosen in order to avoid selection bias.

### Serology, specific antibody detection

*C.burnetii* expresses two antigens, phase II and phase I. In acute Q fever, primarily antibodies against phase II are raised, and their titer is higher than antibodies against phase I. As with most other infections, IgM antibodies appear first.

In chronic forms of the disease, antibodies against phase I are elevated.

When infected, phase II IgG and IgM antibodies are always elevated, and, although declining, they may remain positive for years. A large study from Australia and England found that phase II IgG antibodies persisted after four and 12 years, respectively [16].

The diagnosis of Q fever relies upon serology. In order to determine antibodies agains*t C. burnetii*, we chose a two-step approach. First all samples were screened in a commercial enzyme-linked immunosorbent assay (ELISA). Positive samples from the ELISA were confirmed with an immunofluorescence antibody test (IFA).

The commercial ELISA kit was purchased from Panbio (Queensland, Australia) (cat. no. E-QFB01G and E-QFB01M) and used according to the manufacturer's instructions with one minor modification. Due to low sample volume the samples were not diluted as prescribed in the instructions but same dilution factors were used.

Samples positive for either IgG or IgM antibodies in the ELISA were confirmed with an IFA test from Focus Diagnostics (ca.no. IF0200G and IF0200M). The test was performed according to the instructions provided by the manufacturer, with the following minor modification: due to low amount of sample material, the diluted samples 1∶10 from the ELISA were used to further dilute the samples as described by the manufacturer. The effect of the dilution in the Panbio buffer was tested prior to the use on patient samples and did not show any influence on the results (results not shown).

Also, the IFA cut-off suggested by the manufacturer was not used; since the prevalence of the infection varies between geographic areas, the cut-off suggested by the manufacturer is not necessarily suited for any given area [17]. When reanalyzing the positive ELISA tests using IFA we used the cut-off adjusted to the Danish population as previously described [18].

In the analyses, the titres have been dichotomized in positive and negative according to the Danish cut-off with the inconclusive results categorized as negative.

A sample was considered positive when IgG titres phase I and II against *C. burnetii* were 1∶512 or higher or 1∶1024 or higher, respectively. For IgM a sample was considered positive with a titre of IgM phase I of 1∶128 or higher or IgM phase II of 1∶256 or higher [18].

In acute infection, antibodies against phase II antigens are usually elevated, and a combination of rising antibodies against IgG phase II and IgM usually indicates a present infection.

In a chronic infection, positive antibodies against IgG phase I antigens indicates a possible persisting infection, keeping in mind that diagnosing chronic Q fever requires more than elevated antibodies, including symptoms and supplementary paraclinical tests like PCR and culture of bone marrow.

When investigating the association between Q fever titres and spontaneous abortion we consider IFA to be gold standard. However, as other studies report their results based on ELISA alone, we also report data on ELISA values to ensure comparability with other studies.

All serological analyses were performed according to the manufacturer's instructions in a certified laboratory at Statens Serum Institute, Denmark. Laboratory personnel were blinded for case-status and samples were always analyzed in the same batch of commercial kits.

### Specimens

Blood samples from gestational week 6–12 were collected by the general practitioners. Samples were mailed to the Statens Serum Institut where they were stored at −30°C until assayed.

The final data set included 218 cases and 482 non-cases with interview data covering reproductive history, age and smoking status. For the co-variates, age was split into three categories, women <25 years, 25–35 and above 35 years. Reproductive history was categorized as previous pregnancies ‘yes’ or ‘no’ and smoking status was split into three categories: no smoking, smoking less than 10 cigarettes per day and smoking 10 cigarettes or more per day.

### Statistical analysis

Before the sample sizes were decided, power calculations based on the following assumption was made: The risk of spontaneous abortion in DNBC is about 5%. Using 200 cases and 500 non-cases an odds ratio of 3 could be detected by a power of 80% (significance level: 0.05).

The strength of the association between spontaneous abortion and positive serology was expressed as a crude odds ratio. In an adjusted model we controlled for potential confounding using logistic regression. Maternal age (<25 years, 25–34 years, 35 years+), gravidity (0, 1+) and smoking during pregnancy (0, 1−<10, 10+ cigarettes per day) were *a priori* selected as potential confounders.

Lack of interview data on some participants (table 1) resulted in missing values in the covariates. The missing values were categorized as a separate category for the variable and adjusted analyses were carried out for the entire sample. A subsample of women with complete interview data was also analyzed.

**Table 1:** Distribution of selected maternal characteristics.

	Cases (N = 218)	Non-cases (N = 482)
**Age in years**		
<25	22 (10.09%)	69 (14.32%)
25−<35	148 (67.89%)	358 (74.27%)
35+	48 (22.02%)	55 (11.41%)
Missing	0	0
**Gestational age at recruitment**		
<8 weeks	88 (40.37%)	90 (18.67%)
Week 8–12	115 (52.75%)	224 (46.47%)
Week 12−<16	13 (5.96%)	118 (24.48%)
Week 16+	2 (0.92%)	50 (10.37%)
Missing	0	0
**Gestational age at abortion (cases), delivery or other pregnancy outcome** * **(non-cases)**		
<Week 8	9 (4.13%)	
Week 8–12	107 (49.09%)	
Week 12−<16	78 (35.78%)	
Week 16–22	24 (11.01%)	1 (0.21%)
Week 22–28		2 (0.41%)
Week 28–32		0
Week 32+		479 (99.38%)
Missing	0	0
**Gestational age at blood sampling**		
<Week 6	27 (12.39%)	40 (8.30%)
Week 6–8	87 (39.10%)	131 (27.18%)
Week 8–10	75 (34.40%)	171 (35.48%)
Week 10–12	25 (11.47%)	90 (18.67%)
Week 12–16	4 (1.83%)	38 (7.88%)
Week 16–28	0	12 (2.49%)
Missing	0	0
**Previous pregnancies (N = 617)**		
0	48 (22.02%)	169 (35.06%)
1+	110 (50.46%)	290 (60.17%)
Missing	60 (27.62%)	23 (4.77%)
**Smoking: (N = 611)**		
Non-smokers:	121 (55.5%)	358 (74.27%)
1−<10 cigarettes/day	18 (8.26%)	54 (11.2%)
10+ cigarettes/day	19 (8.72%)	41 (8.51%)
Missing	60 (27.52%)	29 (6.02%)
**No interview data available:**	60 (27.52%)	23 (4.77%)

* Pregnancy outcome: live born singleton, stillbirth, induced abortion after pregnancy week 12 due to illness in the foetus, live born twins.

Quantitative analyses on (log transformed) adjusted ELISA OD-values (optical density values measuring antibody concentrations) were also done using linear regression.

All analyses were carried out using STATA statistical software, version 11.

## Results

A total of 218 pregnancies that ended in miscarriage and 482 non-cases (pregnancies with no spontaneous abortions until 22 gestational weeks) were included. Maternal age was normally distributed in both groups, but higher in the case group (mean 24.7 years (SD: 9.2) vs. mean 22.6 years (SD: 9.7) in the control group. Cases were, on average, recruited at an earlier gestational age than non-cases (8 weeks 5 days (SD2.1) vs. 11 weeks 1 day (SD 3.6)) (Table 1). Among cases, the median age at abortion was 11.7 weeks (range 6.1 to 20.6 weeks). Three (0.6%) non-cases had delivery before gestational week 32. Furthermore, there were more missing interview data for cases than non-cases and a higher proportion of cases had prior pregnancies or were smokers than non-cases (Table 1).

### Detection of antibodies by ELISA

In the initial screening, 11 cases (5.05%) and 29 (6.02%) non-cases were *C. burnetii* positive in ELISA (crude OR: 0.83) (Table 2).

**Table 2:** Crude and adjusted odds ratios for seropositivity for *C.burnetii* in pregnancies ending with miscarriage as compared to control pregnancies.

	Crude OR	Adjusted* OR	95%CI
**ELISA**	0.83	0.94	(0.44–2.02)
**IFA**	0.74	1.19	(0.12–11.70)

* Adjusted for maternal age (with age 25−<35 as reference group), gravidity and smoking.

### Confirmation by IFA

**Figure 1:**
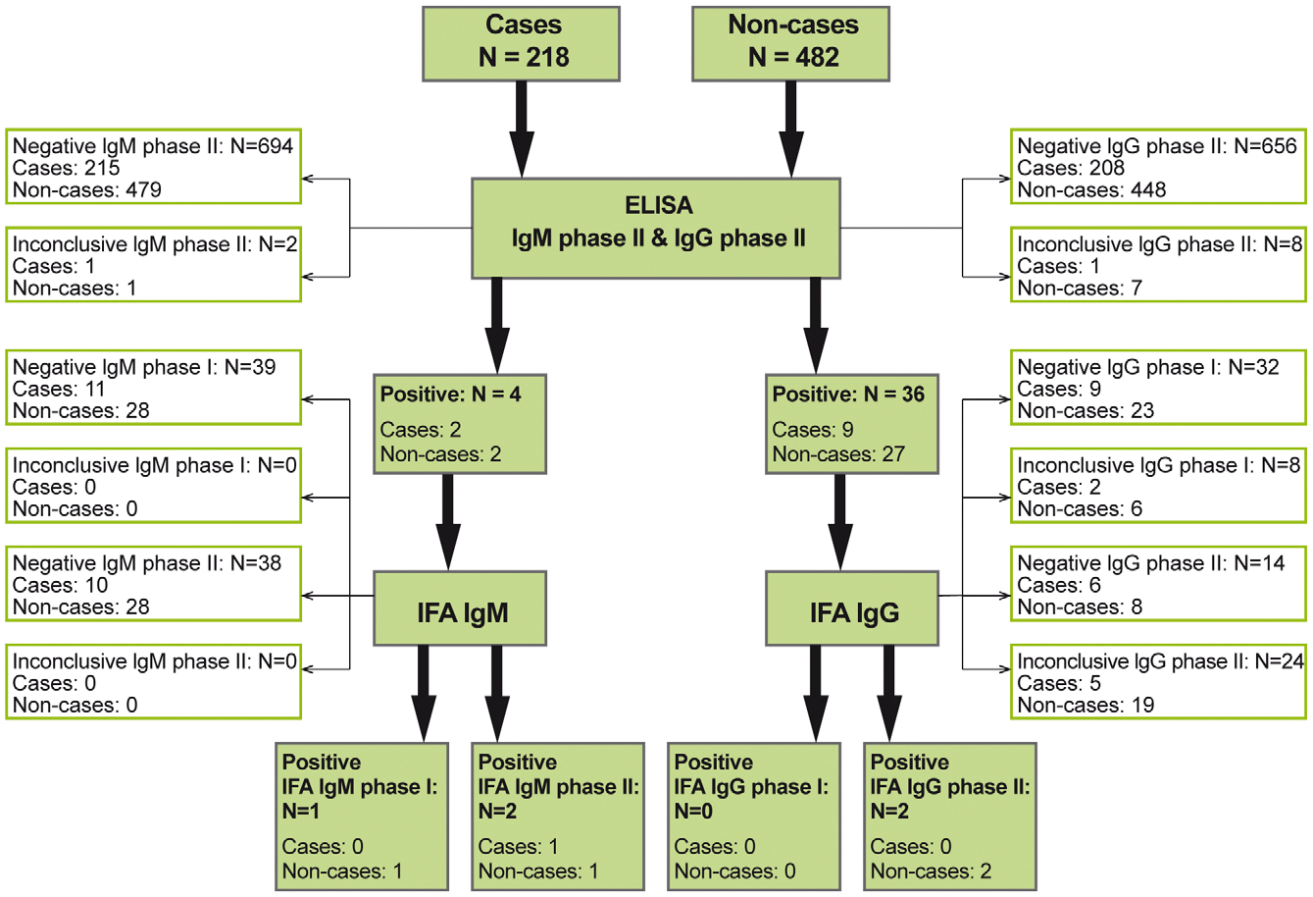
Serologic results: ELISA (using the manufacturer's cut-off) and IFA (using the Danish cut-off) among cases and non-cases. Only assays positive in ELISA were reanalyzed using IFA.

One (0.46%) case was confirmed positive in IFA (IgM phase II positive). For non-cases three (0.62%) were confirmed positive in IFA (IgM phase II positive, IgG phase II positive and IgM phase I as well as IgG phase II positive, respectively) (Figure 1).

Altogether, three women had serologic signs of acute infection, one had signs of a previous infection; none had evident serological signs of chronic infection.

The prevalence of positive vs. negative Q fever titres in IFA was not significantly higher in women with spontaneous abortion before the end of pregnancy week 22 when compared to the control group. The OR for seropositivity for *C.burnetii* in pregnancies ending with miscarriage as compared to control pregnancies was 0.74 (Table 2).

The OR for IFA seropositivity adjusted for a potential confounding effect of maternal age, previous pregnancies and smoking was 1.19; (CI: 0.12–11.70) (Table 2). Adjusted odds ratios were also calculated using the ELISA results. Results for both were similar to the unadjusted estimates (Table 2). OR's adjusted for gestational age at blood sampling were also similar to the unadjusted estimates (results not shown). In a supplementary analysis consisting only of women with complete interview data results remained unchanged (results not shown).

Even though the microimmunofluorescence antibody test (IFA) was regarded as the gold standard in the analyses, supplementary analyses were carried out based on the ELISA results alone. In an age-adjusted, linear regression analysis on adjusted, log-transformed IgG ELISA OD-values, non-cases had 30% higher OD-values than cases, (95% CI: 19%–42%); p<0.0005). Controlling for age in the quantitative comparison did not change the results significantly.

## Discussion

To our knowledge, this is the first population based seroepidemiologic study assessing the association between serologic signs of Q fever and spontaneous abortion. We hypothesized an association between serologic signs of Q fever and spontaneous abortion. Our hypothesis was not confirmed.

Previous case series [11], [12] have concluded high risks of abortions in infected pregnancies. The cases were mainly clinical with Q fever diagnosed in the French National Reference Centre for Rickettsial Diseases during pregnancy, and the findings reported by Raoult et al. could not be reproduced in the present study.

When addressing the Danish population, the selection of diagnosed patients in the French case reports may limit their suitability for assessing the risk of adverse pregnancy outcome among infected, and may give rise to an overestimation of the prevalence of complications.

The Netherlands have recently experienced the world's largest Q fever outbreak [19] and a new Dutch study examined serum samples from 1174 pregnancies with a gestational age of 16 weeks or more from women living in the high-risk area and found no association between positive Q fever serology and adverse pregnancy outcome [20]. However, spontaneous abortion was not the focus of the study.

Outbreaks of Q fever have only been described to occur in small ruminants. In France, goats and sheep have been the source of infection and in the recent Dutch outbreak it was goats. Denmark has never experienced a clinically verified Q fever outbreak and the source of infection is assumed to be cows. A partial explanation to the discrepancy in the existing literature might be different strains of the bacteria with varying virulence and predilection for small ruminants; but this remains unknown [21].

We regard the use of ELISA as well as IFA in the analyses a strength in our study.

A variety of serological methods are available; the Panbio ELISA kit has previously been showed to be superior to other and suitable for large-scale screening [17], [22]. The microimmunofluorescence antibody test (IFA) is regarded as the gold standard [23] due to the fact that it is capable of determining both phase I and II antibodies simultaneously by use of two different antigens on the single sample.

Some countries have defined their own cut-off while others use the cut-off defined by the manufacturer [18].

The IFA cut-off in Denmark is based on 158 anonymous, healthy blood donors from three city areas of Denmark assumed not to have Q fever. Villumsen et al. have chosen a very restrictive cut-off when defining the local baseline in order to obtain a very high specificity [18]. The use of different cut-offs or criteria for the interpretation of serological results hamper the generalisability of serologic results reported in studies from different countries.

Our supplementary analyses based on ELISA values also facilitates comparison to other studies that only use ELISA or use a different IFA cut-off [24].

It is the high positive OD-values in ELISA that are also positive in IFA which illustrates coherence and supports our choice of strategy in the analyses; using ELISA as seroepidemiologic screening with high sensitivity, and regaining specificity in the confirmatory IFA analyses. The inevitable choices included when defining a cut-off are avoided when doing analyses based on quantitative measures, i.e. the quantitative comparison of ELISA OD-values between cases and non-cases independent of cut-offs further supports the conclusion of our results and enhances how analyses based on ELISA values are a useful supplement to provide additional evidence. However, the quantitative comparison of ELISA OD-values also reveals that the average titer values among non-cases are significantly higher than among cases.

While this finding may be coincidental, a possible causal explanation could be that if Q fever is a risk factor for very early abortion, the exposed participants included in this study constitute a robust survived population of pregnancies that survived the most vulnerable period.

Using the manufacturer's IFA cut-off in our study would have resulted in a higher seroprevalence in both groups. However, this would not have affected our conclusion.

When the IFA results were reported, the confidence intervals indicate a low precision, and a larger study would be needed to increase statistical power for detection of smaller effects.

The wide confidence intervals also reflect how the power of the study was negatively affected by the lower than expected seroprevalence among women with spontaneous abortion and by the fact that a sample size of only 218 cases was available.

A recent study used a cohort of Q fever patients to compare serological and PCR results. Although the same IFA method was used, there were large discrepancies in the IFA results between three reference laboratories and the authors proposed development of an international standard of Q fever serological investigation [25].

The discrepancy in the results obtained by different centres compromises our understanding of the natural course of Q fever in pregnancy.

Rather than a stand-alone attempt to change previous risk evaluations, our aim was to perform applicable results and to contribute to the sparse literature on Q fever and adverse pregnancy outcome.

This study has some limitations. Due to the gestational age at enrolment into the cohort, the earliest abortions that constitute the largest proportion of miscarriages were not included in this study. Consequently, we cannot exclude a harmful effect of Q fever infection in very early pregnancy, and furthermore our results may reflect a ‘healthy pregnant population’ due to the fact that the pregnancies have successfully survived through the most vulnerable period. In general, little is known about infections and very early fetal loss. These very early spontaneous abortions are insufficiently registered and thus difficult to approach in research.

When studying causes of spontaneous abortions, adjustment for previous miscarriages is controversial [26]. This is why we chose adjustment for prior pregnancies, regardless of pregnancy outcome. Age is an important factor determining miscarriage risk; adjustment for smoking was justified by the inconsistency of previous findings related to smoking and spontaneous abortion [26].

Decline of especially IgM antibodies could also be a limitation in this study since inclusion of this sample took place between June 1997 and September 2002. However, the four IgM antibody titres positive in ELISA are distributed between Dec 1997 and Feb 2002 and the 36 IgG antibody titres positive in ELISA between Aug 97 and June 02; an Irish study used 20 years old blood samples to evaluate the seroprevalence of IgG phase II antibodies [27].

In conclusion, no association between elevated antibody titres against *C.burnetii* and spontaneous abortion after gestational week 8 was found.

The aetiology of spontaneous abortions remains largely unknown with one third of implanted conceptions failing to survive beyond midpregnancy [26]. Some infections are known to increase the risk of spontaneous abortions, but the role of the specific pathogens has been difficult to demonstrate [28]. The size of this study and the fact that very early spontaneous abortions are not included limit any definite conclusion regarding Q fever infection and risk of early fetal loss.

However, our results suggest that spontaneous abortion associated with *C.burnetii* should not be of great concern among pregnant women.

Regarding directions for future research into *C. burnetii* and spontaneous abortion it would be relevant to study adverse pregnancy outcome in a pregnant population with high-exposed women like veterinarians and female workers with contact to livestock.
